# The Impact of Educational Intervention on Patients of Chronic Obstructive Pulmonary Disease Undergoing Exercise and Medication

**DOI:** 10.7759/cureus.101568

**Published:** 2026-01-14

**Authors:** Jouel Ahamed, Mosammad J Fardoushi, Shohel Ahmed, Tanvir Zoha, Monia Hafiz, Rayhan Sharif, Nazmul Hassan

**Affiliations:** 1 Physical Medicine and Rehabilitation, Dhaka Medical College and Hospital, Dhaka, BGD; 2 Reproductive and Child Health, East View Hospital and Lab, Dhaka, BGD; 3 Physical Medicine and Rehabilitation, Ahsania Mission Cancer and General Hospital, Dhaka, BGD; 4 Physical Medicine and Rehabilitation, Bangladesh Supreme Court Medical Clinic, Dhaka, BGD; 5 Physical Medicine and Rehabilitation, Farazy Hospital Ltd, Dhaka, BGD; 6 Physical Medicine and Rehabilitation, Eastern Medical College and Hospital, Cumilla, BGD

**Keywords:** adherence, bangladesh, copd, exercise therapy, patient education, pulmonary rehabilitation

## Abstract

Introduction

Chronic obstructive pulmonary disease (COPD) is a progressive lung disease marked by airflow limitation, impairing functional capacity and quality of life. Education, exercise, and medication are key components of COPD management, yet evidence on their combined impact remains limited. This study aimed to evaluate the impact of an educational intervention, alongside exercise and medication, on respiratory symptoms, exercise capacity, lung function, and quality of life in COPD patients.

Method

A quasi-experimental study was conducted at Dhaka Medical College Hospital from December 1, 2022, to September 30, 2023. A total of 52 COPD patients aged 40-80 years with mild to moderate disease were enrolled in this study. Patients were purposively allocated into two groups: group A received education, exercise, and medication; and group B received exercise and medication alone. Outcomes measured included dyspnea (mMRC), health status (CCQ), exercise capacity (six-minute walk test (6MWT)), oxygen saturation (SpO₂), and lung function (forced expiratory volume (FEV)₁/forced vital capacity (FVC)). Pre-post changes were analyzed using paired t-tests, and between-group comparisons were made using independent t-tests.

Results

Both groups showed significant improvements from baseline; however, group A demonstrated consistently superior outcomes. After 24 weeks, group A had significantly lower dyspnea scores (1.59 ± 0.50 vs 2.24 ± 0.83; p = 0.001) and better CCQ scores (3.04 ± 0.32 vs 3.31 ± 0.36; p = 0.006). Functional capacity improved more in group A (6MWT: 551.7 ± 32.7 m vs 526.8 ± 37 m; p = 0.01). Oxygen saturation was higher (97.2 ± 0.56% vs 96.2 ± 0.91%; p < 0.001), and pulmonary function improved more notably (FEV₁/FVC: 65.97 ± 2.65 vs 61.78 ± 4.17; p < 0.001) in group A compared to group B.

Conclusion

Educational intervention combined with exercise and medication significantly improved symptoms, functional capacity, oxygenation, and lung function in COPD patients. Structured education programs are recommended as an integral part of COPD management to enhance patient outcomes.

## Introduction

Chronic obstructive pulmonary disease (COPD) is a diverse lung disorder marked by ongoing respiratory symptoms such as breathlessness, coughing, and sputum production caused by lasting structural changes in the airways and/or alveoli, which frequently lead to worsening airflow obstruction over time [1]. This is the fourth leading cause of death globally and is responsible for 3.5 million deaths in 2021, which was about 5% of all deaths. Around 90% of COPD deaths under age 70 occur in low- and middle-income countries (LMICs). It ranks as the eighth leading cause of poor health worldwide [2]. It is a common lung disease marked by progressive airflow limitation and chronic inflammation in the lungs due to harmful particles or gases [2,3]. It is also known as emphysema or chronic bronchitis [3]. It is expected to rise in the future due to an aging population and ongoing exposure to risk factors like tobacco smoke, occupational pollutants, biomass fuel, and air pollution. While tobacco smoking causes over 70% of COPD cases in high-income countries, in LMICs, it accounts for only 30%-40%, with household air pollution being a major contributing factor [4]. Common respiratory symptoms of COPD are shortness of breath (dyspnea), cough, and sputum production [2]. It can cause other health problems like lung cancer, cardiovascular disease, osteoporosis, muscle wasting, and mental health issues [5]. Spirometry is used to diagnose the disease, where a post-bronchodilator forced expiratory volume (FEV)₁/forced vital capacity (FVC) ratio below 0.70 denotes persistent airflow limitation that signifies COPD in patients [2].

Though COPD is not completely recoverable, we can manage it through exercise, medication, education, and public awareness [2]. In 1970, Bass et al. conducted one of the earliest studies on exercise performance in COPD patients with dyspnea during daily activities. After 18 weeks of stationary cycling, patients improved their maximal workload by approximately 85%, and 92% reported their increased daily physical activity [6]. A meta-analysis of randomized trials found that physical exercise improves walking distance (six-minute walk test (6MWT)) and functional capacity in COPD patients, supporting its role in daily life performance and quality of life [7]. However, the intensity of exercise will depend on the preference and tolerance of patients. A study supported 60% utilization of maximum effort instead of 80% in exercise for better physiological benefits [8].

Effective management of COPD requires long-term adherence to medication. It leads a patient toward better health outcomes and improved disease control [9]. Nonadherence to medications may result in disease progression and subsequently reduce the patient's quality of life as well as increase the burden on health systems [10]. A study found that adherence to COPD medication has been significantly impacted by depression, the difficulty of the treatment method, the existence of comorbidities, and therapy adverse effects [11]. However, education initiatives for patients enhance medication adherence, lower hospital admissions, and emergency department visits [12]. Patients with COPD require education and information in order to effectively manage their condition on their own. Their quality of life is improved, and exacerbations are avoided when they possess the skills and motivation to practice self-management. Therefore, a crucial component of treating COPD should be patient education [12].

COPD remains a major cause of disability in Bangladesh, yet most patients struggle with poor symptom control because they lack the knowledge and skills needed for proper inhaler use, exercise routines, and self-management [13]. Medication and exercise alone often fail when adherence is low, and this is especially common in public hospital settings where structured education programs are rarely available [14]. Despite this, very little evidence from Bangladesh evaluates whether adding a simple, low-cost educational intervention can actually improve functional capacity, dyspnea, oxygenation, and lung function.

Therefore, this study aimed to address this gap by conducting an intervention to assess the impact of an educational intervention on patients with COPD undergoing exercise and medication. Its findings can help shape practical rehabilitation strategies and support policymakers in developing patient-centered education programs for COPD care across Bangladesh.

## Materials and methods

Study design

This study followed a quasi-experimental design with two parallel groups. Eligible COPD patients were enrolled consecutively and then purposively allocated into group A (education + exercise + medication) or group B (exercise + medication). Because the intervention involved patient education, participant blinding was not feasible. However, outcome assessors and data analysts were kept unaware of group allocation to minimize measurement bias.

Sample size calculation

We performed the sample size calculation by following 80% power and a confidence level of 99%, based on 6MWT after three months of follow-up [15]. The sample size was calculated to be 48, where each group had 24 patients. Finally, we took 52 patients for increasing accuracy, where 26 patients were included in group A and 26 in group B. The sample size was determined using the following standard formula for comparing two means: \[ n = \frac{(Z_{\alpha} + Z_{\beta})^2 (\sigma_1^2 + \sigma_2^2)}{(\mu_1 - \mu_2)^2} \].

Study setting

The study was conducted from December 1, 2022, to September 2023 at the Department of Physical Medicine and Rehabilitation, in collaboration with the Department of Respiratory Medicine, at Dhaka Medical College Hospital (DMCH), Dhaka, Bangladesh. Participants were selected through consecutive sampling and screened for eligibility by a pulmonologist. We included patients of both genders, aged between 40 and 80 years, residing within 30 km of DMCH, and diagnosed with COPD confirmed by chest X-ray and spirometry, with mild to moderate disease severity. We excluded patients with a history of bronchial asthma, respiratory failure, pneumothorax, pleural effusion, pulmonary tuberculosis, or those who had undergone pulmonary lobectomy. Individuals diagnosed with restrictive lung disease, congestive cardiac failure (CCF), or cardiomyopathy were also excluded. Additionally, patients who had previously participated in a pulmonary rehabilitation program, those with cognitive deficits, or individuals with communicable infectious diseases such as pulmonary tuberculosis or COVID-19 were not eligible for inclusion.

We divided the patients purposively into two groups: group A, which received medical treatments, exercise, and education; and group B, which received medical treatments and exercise. Patients were advised to continue the medical treatment prescribed by the Department of Respiratory Medicine. They were prescribed a 24-week home-based, nonsupervised submaximal exercise program consisting of 30 minutes of activity per day, five days per week, with progress tracked using an evaluation form. Exercises included lower extremity activities like stair climbing and walking, both performed at a self-selected pace and stopped upon reaching 70% of maximum heart rate. Upper extremity training involved weight-lifting and aerobic movements (e.g., arm raises and abductions), performed in sets of 10 repetitions three times daily. Respiratory muscle training incorporated diaphragmatic, pursed-lip, and deep breathing exercises, along with incentive spirometry, all practiced multiple times daily within the same heart rate threshold.

Patient education sessions, conducted by the principal investigator every Thursday from 12:00 to 12:45 pm in the gym room of the physical medicine and rehabilitation (PMR) outpatient department (OPD), lasted 45 minutes and were held every 45 days. Each session included a 15-minute briefing, 10 minutes for self-discussion, and 20 minutes for feedback. Each patient was allowed to bring one attendant, and a Bengali booklet on COPD was provided for easier understanding. Exercises were demonstrated to all patients; however, only group A patients participated in the full educational session, while group B patients were excused afterward. The sessions covered various topics, including the pulmonary rehabilitation (PR) program, lung anatomy and physiology, COPD pathophysiology, clinical features, diagnosis, smoking cessation, immunization, medication use, prevention of exacerbations, nutritional counseling, and psychological support.

Outcome measures

We measured the severity of dyspnea using the mMRC scale; higher mMRC scores are associated with greater health impairment [16]. The Clinical COPD Questionnaire (CCQ) was used to assess health-related quality of life (HRQoL) in individuals with COPD. The CCQ consists of 10 items, each scored from 0 to 6, with higher scores indicating poorer quality of life [16]. For the 6MWT, most studies have used a 30 m corridor, although some have used 20 m or 50 m corridors [17]. We conducted the 6MWT indoors along a flat, straight 15 m corridor due to space limitations to evaluate functional exercise capacity. The corridor was marked at 3 m intervals, with chairs placed at the turnaround points, and a brightly colored pen marked the starting and ending lines on the floor. We also measured peripheral capillary oxygen saturation (SpO₂) using a pulse oximeter; normal levels range between 95% and 100%, levels below 90% indicate shortness of breath, and levels below 70% are life-threatening [18]. Lung function was assessed by spirometry, including FVC, FEV₁, and the FEV₁/FVC ratio for all patients. A portable digital Cosmed micro-Quark spirometer (Manufacturer Catalog #: C09061-01-99) was used for this purpose. COPD was confirmed when the post-bronchodilator FEV₁/FVC ratio was less than 0.70 [19].

Data collection procedure

Patients were followed up at the 24th week, during which a pre-formed exercise evaluation form was used to assess whether they had properly taken prescribed medicine and performed the exercises at home. Data collection was conducted using a pre-designed data collection sheet through face-to-face interviews.

Ethical considerations

The study received ethical approval from the Dhaka Medical College ERC (Memo: ERC-DMC/ECC/2022/320). Participants were clearly informed about the study and gave voluntary written and verbal consent. Confidentiality was ensured, data were used only for research, and withdrawal was allowed at any time. All procedures followed institutional and national ethical standards and the Helsinki Declaration.

Data analysis

**Figure 1:**
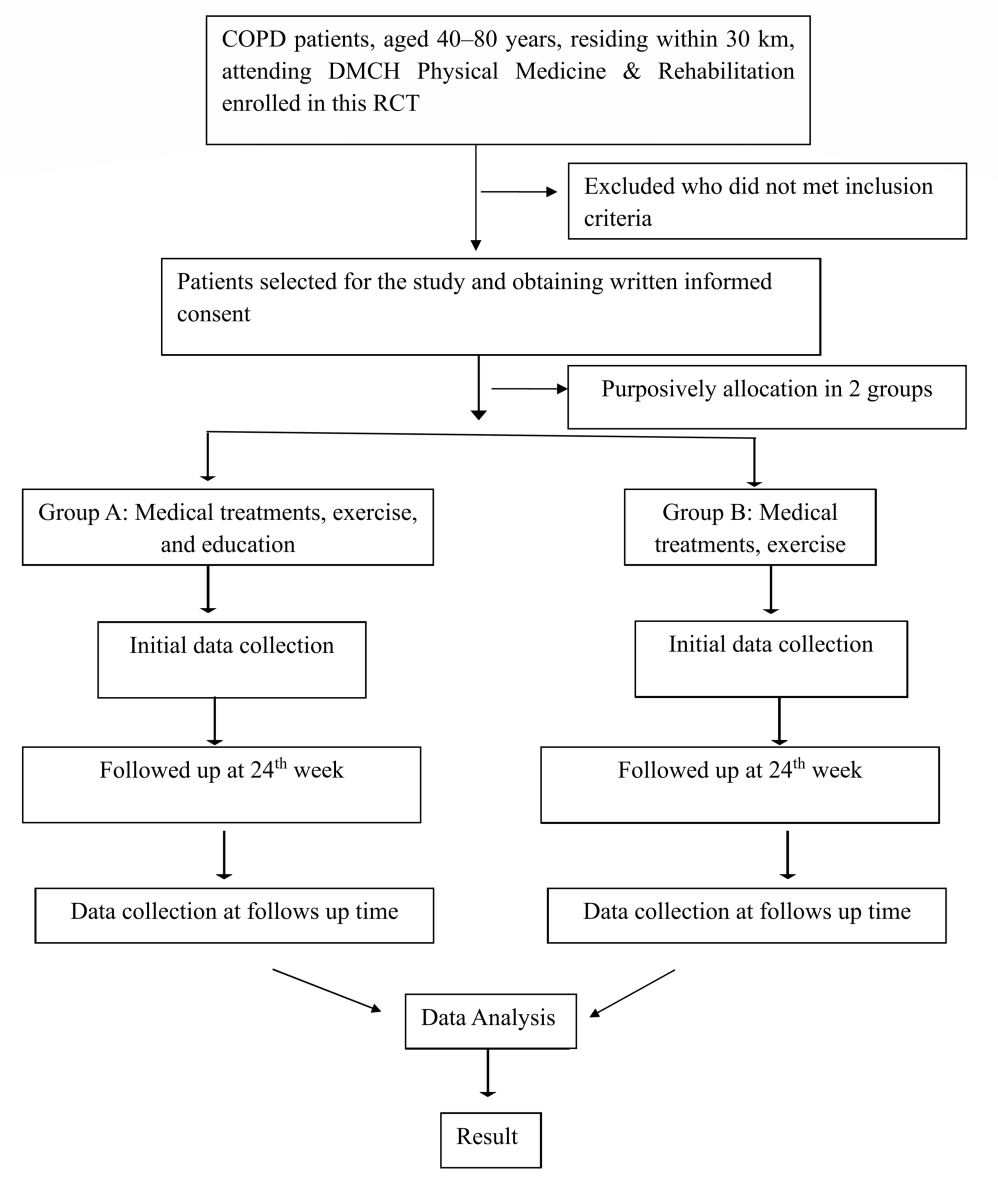
Study flow chart

Data were analyzed using R statistical software (version 4.4.2, R Foundation for Statistical Computing, Vienna, Austria). Continuous variables were summarized as means and standard deviations, and categorical variables were expressed as frequencies and percentages. Between-group comparisons were performed using independent samples t-tests, while within-group pre-post comparisons were conducted using paired t-tests. All statistical tests were two-sided, and a p-value of less than 0.05 was considered statistically significant. Normality of continuous variables was assessed using the Shapiro-Wilk test. For within-group analyses, normality of pre-post change (difference scores) was specifically evaluated. As the normality assumptions were met for the analyzed variables, parametric tests were applied. The overall study flow, including participant screening, group allocation, intervention, and follow-up, is illustrated in Figure 1.

## Results

Table 1 represents the demographic characteristics of the study participants, where the majority of participants were between 51 and 60 years old (22, 42%), followed by 61-70 years (19, 37%), with fewer individuals in the 41-50 years (7, 13%) and 71-80 years (4, 7.7%) age groups. Most participants were males (41, 79%), and only 11 (21%) were females. Regarding educational status, the highest proportion had only primary education (22, 42%), followed by secondary education (15, 29%), while fewer had higher secondary (9, 17%) or above HSC (6, 12%) qualifications. In terms of occupation, the largest group was businessmen (16, 31%), followed by homemakers (11, 21%), day laborers and service holders (both 9, 17%), and teachers (7, 13%). BMI analysis showed that the vast majority (46, 88%) had a normal weight, while four (7.7%) were underweight, and only two (3.8%) were overweight. Smoking history revealed that over half (27, 52%) were previous smokers, 38% were current smokers, and only five (9.6%) were nonsmokers.

**Table 1:** Demographic characteristics HSC: higher secondary certificate; BMI: body mass index Data presented as frequency and percentage

Characteristic	N = 52
Age group	
41-50 years	7 (13%)
51-60 years	22 (42%)
61-70 years	19 (37%)
71-80 years	4 (7.7%)
Gender	
Female	11 (21%)
Male	41 (79%)
Educational status	
Primary	22 (42%)
Secondary	15 (29%)
Higher secondary	9 (17%)
Above HSC	6 (12%)
Occupational status	
Businessman	16 (31%)
Day laborer	9 (17%)
Homemaker	11 (21%)
Service holder	9 (17%)
Teacher	7 (13%)
BMI level	
Underweight	4 (7.7%)
Normal weight	46 (88%)
Overweight	2 (3.8%)
Smoking history	
Nonsmoker	5 (9.6%)
Current smoker	20 (38%)
Previous smoker	27 (52%)

Table 2 presents a comparison of pre- and post-treatment outcomes within group A (n = 26), indicating statistically significant improvements across all measured parameters (p < 0.001). The mean score on the modified mMRC dyspnea scale decreased from 3.33 ± 0.48 to 1.59 ± 0.501, reflecting reduced breathlessness. Similarly, the CCQ score improved from 3.96 ± 0.49 to 3.04 ± 0.32, indicating better clinical status. Physical endurance, assessed by the 6MWT, increased from 499 ± 41.8 m to 551.67 ± 32.7 m, showing enhanced exercise capacity. SpO₂ improved significantly from 94.5 ± 0.58% to 97.2 ± 0.56%, and the FEV1/FVC ratio increased from 56.9 ± 6.24 to 65.97 ± 2.65, indicating better pulmonary function.

**Table 2:** Comparison of pre and post treatment within group A (n = 26) FEV: forced expiratory volume; FVC: forced vital capacity Paired t test was done; *p-value <0.05 was considered statistically significant; **p-value <0.001 was considered statistically significant

Characteristic	Mean ± SD	t	P-value
Medical Research Council (mMRC) dyspnea scale			
Pre-treatment	3.33 ± 0.48	-12.78	<0.001**
Post-treatment	1.59 ± 0.501		
Clinical COPD Questionnaire (CCQ)			
Pre-treatment	3.96 ± 0.49	-8.01	<0.001**
Post-treatment	3.04 ± 0.32		
Six-minute walk test (6MWT)			
Pre-treatment	499 ± 41.8	5.06	<0.001**
Post-treatment	551.67 ± 32.7		
Oxygen saturation (SpO₂)			
Pre-treatment	94.5 ± 0.58	17.08	<0.001**
Post-treatment	97.2 ± 0.56		
FEV_1_/FVC ratio			
Pre-treatment	56.9 ± 6.24	6.82	<0.001**
Post-treatment	65.97 ± 2.65		

A comparison of various clinical parameters before and after treatment in group B (n = 26), demonstrating statistically significant improvements across all measures (p < 0.001). The mean mMRC dyspnea scale score decreased from 3.24 ± 0.597 to 2.24 ± 0.831, indicating a notable reduction in perceived breathlessness. The Clinical COPD Questionnaire (CCQ) score improved from 3.9 ± 0.262 to 3.31 ± 0.359, reflecting better overall health status and symptom control. Functional capacity, as measured by the 6MWT, increased from 484 ± 45.6 meters to 527 ± 37 meters, suggesting enhanced exercise tolerance. SpO₂ also improved from 94.4% ± 0.507 to 96.2% ± 0.913, indicating better oxygenation. Additionally, the FEV1/FVC ratio rose from 54.1% ± 6.04 to 61.78% ± 4.17, suggesting improved pulmonary function (Table 3).

**Table 3:** Comparison of pre and post treatment into group B (n = 26) Paired t test was done; *p-value <0.05 was considered statistically significant; **p-value <0.001 was considered statistically significant

Characteristic	Mean ± SD	t-value	p-value
Medical Research Council (mMRC) dyspnea scale			
Pre-treatment	3.24 ± 0.597	4.98	<0.001**
Post-treatment	2.24 ± 0.831		
Clinical COPD Questionnaire (CCQ)			
Pre-treatment	3.9 ± 0.262	6.77	<0.001**
Post-treatment	3.31 ± 0.359		
Six-minute walk test (6MWT)			
Pre-treatment	484 ± 45.6	-3.73	<0.001**
Post-treatment	527 ± 37		
Oxygen saturation (SpO₂)			
Pre-treatment	94.4 ± 0.507	-8.79	<0.001**
Post-treatment	96.2 ± 0.913		
FEV_1_/FVC ratio			
Pre-treatment	54.1 ± 6.04	-5.34	<0.001**
Post-treatment	61.78 ± 4.17		

Table 4 represents the post-treatment comparison between group A and group B, revealing statistically significant improvements in all measured outcomes for group A. Group A demonstrated a lower mean score on the mMRC dyspnea scale (1.59 ± 0.501) compared to group B (2.24 ± 0.831), indicating less perceived breathlessness (p = 0.001). Similarly, group A showed better health status on the Clinical COPD Questionnaire with a lower mean score (3.04 ± 0.32 vs. 3.31 ± 0.359, p = 0.006). Functional capacity, as assessed by the 6MWT, was higher in group A (551.67 ± 32.7 m) than in group B (526.84 ± 37 m, p = 0.01). SpO₂ levels were also significantly higher in group A (97.2 ± 0.557%) compared to group B (96.2 ± 0.913%, p < 0.001). Additionally, the FEV1/FVC ratio, a key indicator of pulmonary function, was better in group A (65.97 ± 2.65) than in group B (61.78 ± 4.17, p < 0.001).

**Table 4:** Comparison of post treatment between group A and group B (n = 26) FEV: forced expiratory volume; FVC: forced vital capacity; COPD: chronic obstructive pulmonary disease Independent t-test was done; *p-value <0.05 was considered statistically significant; **p-value <0.001 was considered statistically significant

Characteristic	Mean ± SD	t-value	p-value
Medical Research Council (mMRC) dyspnea scale			
Group A	1.59 ± 0.501	-3.42	<0.001**
Group B	2.24 ± 0.831		
Clinical COPD Questionnaire			
Group A	3.04 ± 0.32	-2.86	0.006*
Group B	3.31 ± 0.359		
Six-minute walk test			
Group A	551.67±32.7	2.56	0.010*
Group B	526.84±37		
Oxygen saturation			
Group A	97.2±0.557	4.77	<0.001**
Group B	96.2±0.913		
FEV_1_/FVC ratio			
Group A	65.97±2.65	4.32	<0.001**
Group B	61.78±4.17		

## Discussion

This study evaluated the impact of a structured educational intervention on clinical and functional outcomes in patients with COPD receiving exercise and medication. The results showed that the educational intervention group achieved significantly greater improvements in dyspnea, health-related quality of life, exercise capacity, SpO₂, and pulmonary function compared to the control group.

At baseline, both groups had similar demographic and clinical characteristics, reflecting the typical COPD population seen in clinical practice: predominantly male, middle-aged to older adults, with moderate education levels, and a high prevalence of smoking history. These factors likely contributed to the generally low treatment adherence and poor clinical outcomes observed before the intervention, similar to previous reports that adherence to chronic respiratory therapy remains low in real-world settings and is estimated at only about 50% globally [20]. The COVID-19 pandemic may also have exacerbated this trend, as patients avoided hospital visits and often discontinued treatment due to logistical difficulties or fear of infection [21]. This underscores the importance of accessible, home-based educational strategies to improve adherence in such challenging contexts.

The intervention group showed significantly better outcomes across all measured domains compared to the control group. Reductions in dyspnea and improvements in CCQ scores observed in our study align with findings by prior research demonstrating that education combined with rehabilitation improves symptom control and quality of life in COPD patients [12]. Similarly, the marked increase in exercise capacity, reflected by longer 6MWT distances, is consistent with the benefits of pulmonary rehabilitation reported in earlier studies [22,23]. Notably, our intervention also improved oxygenation and lung function, suggesting that improved adherence to inhaler therapy and regular performance of breathing exercises may reduce air trapping and enhance ventilation efficiency. Prior study found these similar findings, and they stated that improved adherence to inhaler therapy and regular performance of breathing exercises significantly enhance lung function (e.g., FEV1) and exercise capacity and reduce dyspnea in COPD patients, contributing to improved quality of life [24,25].

Several factors likely explain the positive outcomes observed. The education sessions delivered by nurses provided patients with disease knowledge, skills in inhaler technique and breathing exercises, and strategies for self-management, which likely improved confidence, motivation, and adherence [26]. Regular follow-up calls and video materials may have reinforced learning and helped maintain engagement. This comprehensive, culturally adapted, and nurse-led approach differs from prior interventions, such as pharmacist-led programs that focused solely on medication adherence and reported more modest benefits [27]. The personal interaction and continuous support offered by nurses in our study appear to have been particularly effective in addressing misconceptions and sustaining behavioral change [28].

Our findings are consistent with previous randomized trials and systematic reviews that have shown the effectiveness of education-based interventions in improving adherence and clinical outcomes in COPD [12,29]. For example, Bourbeau et al. demonstrated that structured self-management programs for COPD patients significantly reduced hospitalizations for exacerbations and improved health-related quality of life [29]. Similarly, our results align with those of Zwerink et al., who found that education enhanced disease knowledge, self-efficacy, and adherence [12]. Education may improve patients’ understanding of symptom control and early exacerbation management, leading to better adherence and, ultimately, better clinical outcomes.

This study has several important limitations. The sample size was small and derived from a single tertiary care center, which may limit the generalizability of the findings. The follow-up period was relatively short, precluding assessment of long-term adherence, sustainability of improvements, and delayed clinical outcomes. Adherence to home-based exercise and educational components relied on patient self-report, introducing the possibility of recall and reporting bias. Blinding of participants was not feasible due to the educational nature of the intervention. Furthermore, because group allocation was purposive rather than randomized, baseline differences between groups may not have been fully accounted for, and thus, between-group comparisons should be interpreted with caution. Future studies should include larger, multicenter cohorts with extended follow-up to evaluate sustained benefits. Incorporation of objective adherence measures, such as digital activity monitoring or supervised tele-rehabilitation, may reduce self-reporting bias. Additional evaluation of cost-effectiveness and patient satisfaction would further support the integration of structured educational interventions into routine COPD care.

## Conclusions

Educational intervention added to exercise and medication produced greater gains in dyspnea relief, functional capacity, oxygenation, and lung function than exercise and medication alone, showing it to be safe and associated with greater improvement as an adjunct in COPD care. Its advantages include improved adherence, better symptom control, and enhanced self-management. Larger multicenter studies with longer follow-up are needed to confirm these outcomes and refine implementation strategies.
